# A Remarkable Instance of Longevity

**Published:** 1886-03

**Authors:** 


### A Remarkable Instance of Longevity.

The *Popular Science News* for December publishes a portrait of Madame Girard, a native of the town of Saint-Just-de-Claix, in France. According to the parish records, she was born on the 16th of March, 1761, and is therefore a hundred and twenty-four years old. Like the traditional old lady of all countries, she can sew without using spectacles, and her hearing-powers are still good. Her memory, however, is very much impaired; and she remembers little of the events of her long life. She is still active, and carries her own wood and water, besides doing all the work of her modest house.

Her diet is quite simple, and consists principally of vegetables, with little or no meat. After every meal she indulges in glass of wine or brandy. The *News* goes on to say: "Our contemporary, *La Nature*, to which we are indebted for the portrait, gives a list of persons, in France and elsewhere, who have exceeded the age of a hundred years. It is surprisingly large, and includes the well-known Thomas Parr, who died at the age of a hundred and fifty-two years, and a Frenchman named Priou, who died in 1838, in his hundred and fifty-eighth year.

It should be remembered that in England and Europe the records of births are much more carefully kept than in this country, and these remarkable ages appear to be well authenticated. Census returns show that in France, during the years 1824 to 1837, there were between a hundred and eleven and a hundred and seventy-five centenarians; while in Russia, in 1838, there was the surprising number of twelve hundred and thirty-eight. They also show that the average length of life is constantly increasing, and the time may yet come when persons a hundred years old will excite no more curiosity than one of eighty years at the present time."

